# Effect of Row Spacing and Seeding Rate on Russian Thistle (*Salsola tragus*) in Spring Barley and Spring Wheat

**DOI:** 10.3390/plants10010126

**Published:** 2021-01-09

**Authors:** Judit Barroso, Nicholas G. Genna

**Affiliations:** Columbia Basin Agricultural Research Center, Oregon State University, Adams, OR 97810, USA; nicholas.genna@oregonstate.edu

**Keywords:** crop competition, cultural management, rainfall, rain-fed agriculture, seed production, weed suppression, weed density

## Abstract

Russian thistle (*Salsola tragus* L.) is a persistent post-harvest issue in the Pacific Northwest (PNW). Farmers need more integrated management strategies to control it. Russian thistle emergence, mortality, plant biomass, seed production, and crop yield were evaluated in spring wheat and spring barley planted in 18- or 36-cm row spacing and seeded at 73 or 140 kg ha^−1^ in Pendleton and Moro, Oregon, during 2018 and 2019. Russian thistle emergence was lower and mortality was higher in spring barley than in spring wheat. However, little to no effect of row spacing or seeding rate was observed on Russian thistle emergence or mortality. Russian thistle seed production and plant biomass followed crop productivity; higher crop yield produced higher Russian thistle biomass and seed production and lower crop yield produced lower weed biomass and seed production. Crop yield with Russian thistle pressure was improved in 2018 with 18-cm rows or by seeding at 140 kg ha^−1^ while no effect was observed in 2019. Increasing seeding rates or planting spring crops in narrow rows may be effective at increasing yield in low rainfall years of the PNW, such as in 2018. No effect may be observed in years with higher rainfall than normal, such as in 2019.

## 1. Introduction

Russian thistle (*Salsola tragus* L.) is a summer annual broadleaf weed that is widely distributed throughout the western United States [1]. A healthy, well-established winter wheat crop is competitive with Russian thistle, whereas spring crops such as spring wheat (*Triticum aestivum*) or spring barley (*Hordeum vulgare*) suffer from competition-associated yield loss that is exacerbated during dry years [2]. In addition to potential crop yield loss, Russian thistle can reduce the yield of the subsequent crop through rapid postharvest regrowth and soil water depletion [3,4]. Individual Russian thistle plants were previously shown to use 70 L of soil water during spring wheat development and an additional 170 L of soil water following harvest [3]. 

A majority of wheat produced in the inland Pacific Northwest (PNW) relies on non-selective herbicides or tillage to control weeds following harvest and during fallow. The ubiquitous glyphosate use in this region is selecting for glyphosate-resistance in major agronomic weeds [5]. Russian thistle is one weed that is a persistent issue in low and intermediate precipitation zones of the PNW that has recently been identified as glyphosate-resistant in Oregon, Montana, and Washington [6,7]. Farmers in the PNW need more integrated strategies to control Russian thistle and other weeds to prolong the usefulness of herbicides and the sustainability of wheat production systems in the region. Diversifying the common winter wheat–fallow cropping system in the inland PNW with the introduction of a spring crop could help reduce herbicide pressure and provide opportunities to control winter annuals, in addition to other benefits [8,9]. However, spring crops could facilitate the increase in summer annuals such as Russian thistle.

Reducing crop row spacing or increasing crop seeding rates are two cultural management practices that can increase crop competition and weed suppression in cereals [10,11,12]. For example, increasing spring barley planting density from approximately 63 to 161 plants m^−2^ increased yield and decreased rigid ryegrass (*Lolium rigidum*) tiller number [13]. Furthermore, for example, decreasing durum winter wheat inter-row spacing from 25 to 5 cm provided significantly greater control of various weed species in two cultivars, with a significant increase in grain yield in one cultivar [14]. 

However, a higher seeding rate or reduced row spacing is not always beneficial and may have no effect on weed control. Increasing durum winter wheat seeding density from 190 to 570 seeds m^−2^ did not significantly reduce weed biomass or increase yield [14]. Similarly, Kolb et al. [15,16] found similar spring wheat and spring barley yields between different crop densities ranging from 200 to 600 plants m^−2^ and crop row widths ranging from 11 to 23 cm with *Sinapis alba* weed pressure. 

Modifying row spacing or seeding rate is an effective cultural management practice with most crops and weed species, although there have been reported cases where it was not effective. Various reasons are employed in instances where weed control is insufficient, including soil quality, year-to-year climate variation, crop cultivar performance, or weed species of interest. Research is necessary to evaluate the efficacy of reducing row spacing and increasing seeding rates to reduce Russian thistle density in spring crops. The objective of this research was to determine how seeding rate and/or row spacing affect Russian thistle emergence, mortality, plant biomass, and seed production within spring barley and spring wheat crops. 

## 2. Results and Discussion

**Figure 1:**
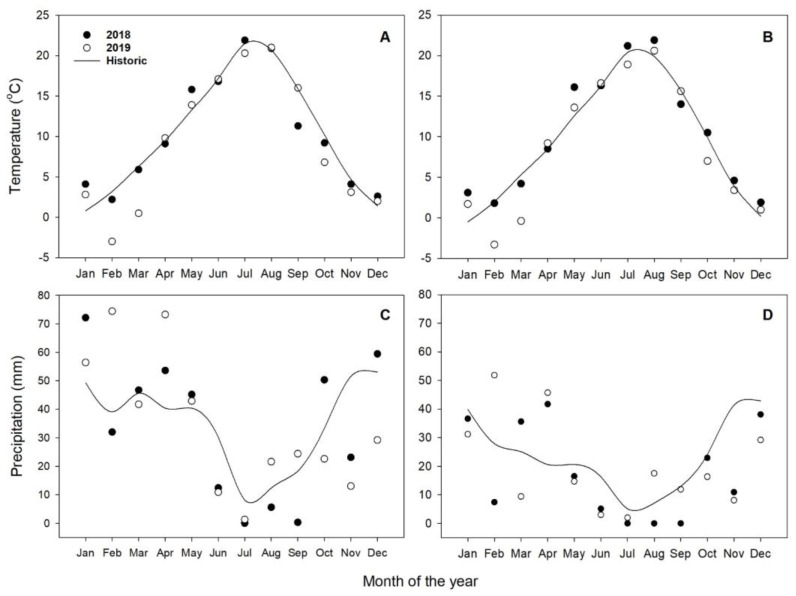
Annual temperature and precipitation for Pendleton (**A**,**C**) and Moro (**B**,**D**) in 2018 (black circles) and 2019 (open circles). Historic data (solid line) date back to 1932 in Pendleton and 1897 in Moro.

Total precipitation received at the Pendleton site from seeding to harvest was 111 and 150 mm in 2018 and 2019, respectively, and 63 and 83 mm in Moro during 2018 and 2019, respectively. The average total rainfall for Pendleton in 2018 and 2019 was 401 and 412 mm, respectively, while the average total rainfall for Moro in 2018 and 2019 was 215 and 241 mm, respectively (Figure 1).

### 2.1. Russian Thistle Emergence and Mortality

Russian thistle emergence differed between years, sites, crops, and their interaction (Table 1). Pendleton, in 2018, had the highest emergence (55.5 plants m^−2^), and Moro, in 2019, had the lowest emergence (9.1 plants m^−2^). Higher rainfall was likely a primary driver of higher emergence in Pendleton compared to Moro. In Lind, WA, an area that receives approximately 248 mm year^−1^ of rainfall, Russian thistle density in spring wheat was 2.2 times lower in two years that received an average rainfall of 215 mm compared to a year that received 356 mm when averaged across treatments [2].

Russian thistle density decreased in all plots during the growing season. Plant mortality differed between crops and the interaction between year × site or year × crop (Table 1). Russian thistle mortality was higher in spring barley (48%) compared to spring wheat (37%). Similarly, Borger et al. [17] found that barley suppressed *L. rigidum* plant density and biomass to a greater extent compared to wheat in two of three sites in one year. These authors also demonstrated that photosynthetically active radiation was lower in barley in the inter-row space, which may have contributed to greater *L. rigidum* suppression. Barley may be more competitive than wheat due to greater early biomass, higher leaf area index throughout development, and potential allelopathic activity [18,19,20]. This research supports the broad notion of barley’s competitiveness and offers novel support for greater competitiveness with Russian thistle compared to spring wheat. 

Russian thistle mortality was highest in spring barley in 2018 (59%) and lowest in spring wheat in 2019 (35%). Mortality was similar among Pendleton 2018 (51%), Moro 2018 (47%), and Moro 2019 (45%), while mortality was lowest in Pendleton 2019 (26%). Russian thistle mortality rate was at an average of 37% in spring wheat across sites and years, which was much higher than the 8% mortality reported by Young [4] in spring wheat across two years. It is not currently known how Russian thistle mortality is related to shading, emergence time, or emergence position (i.e., inter-row vs. in-furrow). Further research is necessary to address this knowledge gap. 

Higher crop seeding density reduced Russian thistle emergence in spring barley in Pendleton 2019 and increased mortality in spring wheat in Moro 2018. Russian thistle emergence did not differ between row spacing treatments in spring barley in either year, whereas emergence was different in spring wheat in 2019 at both sites with a location-dependent effect (Table 2). Narrow row spacing decreased Russian thistle emergence in Moro 2019 when the crop was competitive due to higher rainfall. To the contrary, wide row spacing produced lower Russian thistle emergence in Pendleton 2019 when the crop had a significant infestation of netseed lambsquarter (*Chenopodium berlandieri*) that was removed in mid-June. It is possible that the presence of netseed lambsquarter in the trial area could have reduced Russian thistle emergence due to higher competition in the wide row spacing treatment. Higher soil disturbance in narrow rows may have also favored Russian thistle emergence. Russian thistle emergence has been observed to increase in disturbed soil compared with no tilled soil [21]. Row spacing did not affect Russian thistle mortality.

### 2.2. Effects on Russian Thistle Seed Production and Plant Biomass

There was a significant three-way interaction between year, site, and crop with respect to Russian thistle seed production (Table 1). Russian thistle plants produced the greatest number of seeds in spring wheat in Moro 2019, averaging 2078 seeds plant^−1^. Russian thistle plants produced the fewest seeds in spring barley and spring wheat in Pendleton during 2019 and in spring barley in Moro during 2018, averaging approximately 385 seeds plant^−1^. 

**Figure 2:**
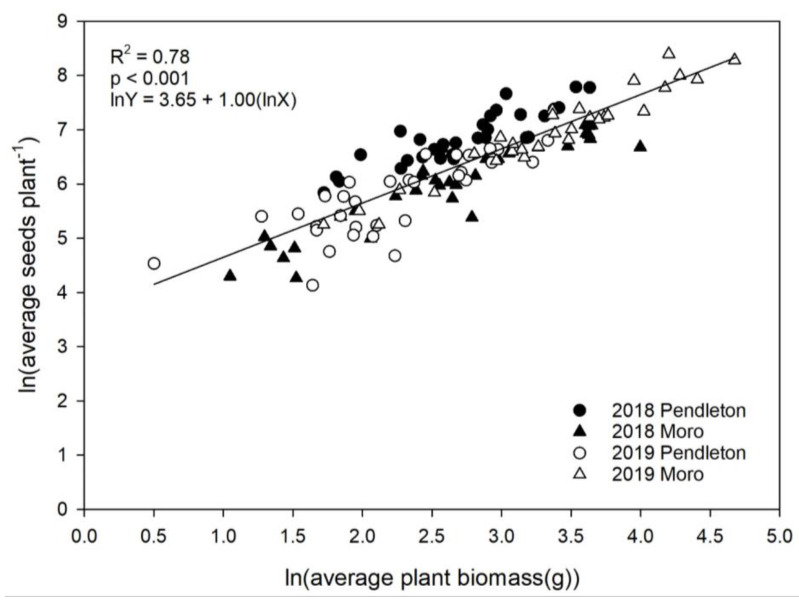
Relationship between average Russian thistle plant biomass (g) and Russian thistle seed production (seeds plant^−1^) across sites and years. Solid line represents the average across sites and years.

Plant biomass followed a similar pattern to seed production with a significant three-way interaction between year, site, and crop (Table 1). Russian thistle plants were largest in spring wheat in Moro 2019 (46.8 g) and smallest in spring barley in Pendleton 2019 (6.9 g). Russian thistle seed production increased linearly with plant biomass and was similar among sites and years (analysis of covariance (ANCOVA) F_3,119_ = 0.655. *p* = 0.581; Figure 2). This indicates that larger Russian thistle plants have a greater likelihood of producing more seeds irrespective of location and year. Russian thistle grows rapidly following harvest, and final plant size may depend on size at crop harvest [3,4]. Young [4] demonstrated that Russian thistle growing in winter wheat accumulated less biomass by harvest compared to Russian thistle growing in spring wheat. In this study, biomass was not measured until approximately two months after harvest, but Russian thistle biomass was significantly lower in spring barley (14 g) compared to spring wheat (26 g) (Table 1).

Row spacing and seeding rate did not affect Russian thistle seed production or plant biomass in either year (Table 2). The lack of a broad effect of row spacing or seeding rate on Russian thistle seed production and plant biomass mirrored the weak effects observed on Russian thistle emergence and mortality. In contrast, Paynter and Hills [13] demonstrated a linear decrease in *L. rigidum* tiller number with increasing spring barley planting density. Borger et al. [22] also demonstrated declining *L. rigidum* seed number m^−2^ in various crops, including barley and wheat, as row spacing treatments declined from 36 to 9 cm. Unlike *L. rigidum*, however, Russian thistle does not senesce by harvest, continuing to grow until a killing frost [3]. 

### 2.3. Effects on Crop Yield

The combination of year, site, and crop type produced a significant three-way interaction when yield with seeded Russian thistle was examined (Table 1). In 2018, the wheat yield in Pendleton was 3.7 times higher than the wheat yield in Moro at 3941 and 1060 kg ha^−1^, respectively. Similarly, the barley yield in Pendleton was 4.1 times higher than the barley yield in Moro during 2018 at 5611 and 1375 kg ha^−1^, respectively. The relationship reversed in 2019 for both crops with 1.5 and 1.6 times higher wheat and barley yields, respectively, in Moro compared to Pendleton. Pendleton is typically higher yielding than Moro due to its location in a higher rainfall zone. The lower yield in Pendleton in 2019 may have been due to the trial following winter wheat instead of fallow, as occurred in 2018. The trial in Moro 2019 also followed spring barley instead of winter wheat, as occurred in 2018. This combination of events likely contributed to the higher yield in Moro during 2019. 

Spring barley yield was 1.4 times higher than spring wheat yield in both years (Table 1). Barley yields significantly more produce than wheat in the PNW and in other areas due to greater above-ground dry mass [19,23]. Spring barley yield was also significantly higher in 2019 than in 2018, whereas spring wheat yield was similar in 2018 and 2019. Higher spring barley yield in 2019 was likely driven by higher spring precipitation that year.

Narrow row spacing did not produce higher yields than wider row spacing treatments. For example, spring barley planted in wider rows produced higher yields than the narrow row spacing treatment in Moro 2018. In contrast, yield tended to increase with higher crop seeding density in spring barley and spring wheat, except for Moro 2019 where spring barley yield was higher in the lower seeding density treatment (Table 2). Previous research has demonstrated that yield increases when crops are sown in narrower rows or at higher seeding rates with weed pressure since greater crop competition suppresses weed growth and biomass [24,25]. In this study, however, higher yield with a higher crop density was independent of weed presence, since yield in Russian-thistle-free sub-sub-plots was similar to infested sub-sub-plots in 2019 (Table 3). This demonstrates that a Russian thistle density of 15 plants m^−2^ or lower may not have a significant impact on spring wheat or spring barley yield in a year with higher growing season rainfall than average. Similarly, Young [2] demonstrated no effect of Russian thistle on spring wheat yield in one year with high early growing season rainfall, while two other years with less rainfall were affected by Russian thistle pressure. Unfortunately, Russian-thistle-free yield data were not obtained in 2018, precluding broad conclusions about effects of Russian thistle on yield in a lower rainfall year, except for observations that emergence was higher in 2018.

## 3. Materials and Methods

### 3.1. Location

A two-year field experiment was established in Umatilla and Sherman counties located in Oregon during 2018 and 2019. The Umatilla site was located at the Columbia Basin Agricultural Research Center (CBARC) in Adams, Oregon, hereafter referred to as the Pendleton site. The Sherman site was located at CBARC in Moro, Oregon, hereafter referred to as the Moro site. Both sites are rain-fed sites. The soil at the Pendleton site was a Walla Walla silt loam (8% clay, 27% sand, and 65% silt) with 2.3% organic matter and a pH of 5.4. The soil at the Moro site was a Walla Walla silt loam (7% clay, 30% sand, and 63% silt) with 1.2% organic matter and a pH of 6.6. The Pendleton site is located in an intermediate precipitation zone while the Moro site is located in a low rainfall zone of the PNW. Long-term average precipitation at the Pendleton site is 421 mm year^−1^ while the Moro site receives 287 mm year^−1^. Fertilization was applied in all sites following standard recommendations for the region [26]. All sites were managed following conventional tillage practices, except for the site in Moro 2018 which was a no-till site. The Pendleton site used in 2018 was fallow in 2017 and the Pendleton site used in 2019 had winter wheat in 2018. The Moro site used in 2018 had winter wheat in 2017 and the Moro site used in 2019 had spring barley in 2018.

### 3.2. Experimental Design

The experimental design in 2018 was a split-plot randomized complete block design with four replications. Row spacing (18 or 36 cm) was the main plot factor and seeding rate (73 or 140 kg ha^−1^) was the sub-plot factor. The main plot was doubled to accommodate two crop types (spring wheat “WB6341” or spring barley “Champion”). Both crops were included in the same experimental area to guard against a soil type or field topography effect on the crop and Russian thistle development. The main plot was 1.7 m × 36.6 m while the sub-plot was 1.7 m × 9.15 m. Each main plot had then four sub-plots (spring wheat at 73 and 140 kg ha^−1^ and spring barley at 73 and 140 kg ha^−1^). Crop type and seeding rate were randomized within main plots. Each main plot was separated by a 1.7-m alley. Russian thistle seed was spread by hand at a rate of 0.43 g m^−2^ before seeding the crops with a Hege 9-row drill on April 19 in the Pendleton site and April 20 in the Moro site.

The experimental design in 2019 was a split-split-plot randomized complete block design with four replications. Main plot size increased to 1.7 m × 48.8 m and sub-plot size increased to 1.7 m × 12.2 m. The first 6.1 m of the sub-plot were hand-seeded with Russian thistle while the second 6.1 m of the sub-plot remained Russian-thistle-free. Crop type, row spacing, and seeding rate treatments were the same as in 2018. Experiments were seeded on 19 and 22 April 2019 at the Pendleton and Moro sites, respectively. In the Pendleton site, sub-sub-plots without seeded Russian thistle were sprayed with pyrasulfotole + bromoxynil herbicide (Huskie^®^, Bayer CropScience, Chesterfield, MO, USA) and sub-plots seeded with Russian thistle were hand weeded on 14 June 2019 to control a weed infestation of netseed lambsquarter that occurred in 2019 at that site. The lambsquarter was fully controlled in a few days after its detection. For all the other sites, the weed control before the crop seeding and Russian thistle seed spreading (by tillage or by a non-residual, non-selective herbicide) was enough to maintain the plots weed-free except for the Russian thistle infestation. 

### 3.3. Data Collection

Russian thistle evaluations were conducted by placing a sampling frame (0.5 m × 0.5 m = 0.25 m^2^) in six random locations within each sub-plot in 2018. In 2019, larger sampling frames (1 m × 0.5 m = 0.5 m^2^) were used to sample six random locations due to a lower Russian thistle infestation that year. Post-seeding evaluations (hereafter referred to as emergence evaluations) at each location were conducted on 22–23 May in 2018 and 25–26 June in 2019. Spring wheat and spring barley were harvested on 25 and 31 July 2018 at the Moro and Pendleton sites, respectively. Harvest in 2019 was on 20 and 21 August in Pendleton and Moro sites, respectively. Yield data were determined per sub-plot in 2018 and sub-sub-plot in 2019.

Russian thistle plants were harvested on 15 and 19 October 2018 and 25 October and 1 November 2019 at the Pendleton and Moro sites, respectively. Five plants were randomly removed from each sub-plot in 2018 and sub-sub-plot in 2019, placed inside paper bags, and moved to a greenhouse to be processed at a later date. Russian thistle plants were first weighed to determine dry biomass and then hand-threshed. The processed material was passed through a series of sieves to obtain a seed and chaff mixture. The seed and chaff mixture was weighed and seed number was determined from an approximate 0.5-g sample of the mixture. Seed number per plant was determined by dividing the seed number in each sample by 0.5 and then multiplying the result by the total weight of the seed and chaff.

### 3.4. Statistical Analyses

Linear mixed models (LMMs) were used to study the effect of year, site, crop type, row spacing, and seeding rate on Russian thistle emergence, mortality, seed production, plant biomass, and crop yield. Due to the high number of independent variables, two analyses were conducted separately. The first analysis included year, site, and crop type effects on Russian thistle emergence, mortality, plant biomass, seed production, and crop yield. The second analysis included row spacing and seeding density effects on the aforementioned variables. Fixed effects in the first analysis were year, site, and crop, while seeding density and row spacing were the fixed variables in the second analysis. Replications in the first analysis were the random effect, while replications nested within row spacing were the random effect in the second analysis. RStudio v.1.2 software (RStudio Team, Boston, MA, USA) was used for all analyses. The least-square means function (lsmeanS) in R studio was used for mean separation in the LMMs, specifying Tukey’s test.

Linear regression was used to evaluate the relationship between Russian thistle plant biomass and seed production among sites and years. A natural logarithm transformation was applied to both axes to satisfy normality. Differences in slopes between regression models were assessed with analysis of covariance (ANCOVA), specifying the ANOVA_test function in the rstatix package of R studio.

Mean crop yield with and without Russian thistle pressure for each location and crop in 2019 was assessed with a one-way analysis of variance and means were compared with Tukey’s test. All data were checked graphically for normality assumptions before conducting the analyses. All figures were created in SigmaPlot v.14 (Systat Software, San Jose, CA, USA).

## 4. Conclusions

The absence of a broad effect of row spacing, seeding rate, or their interaction on Russian thistle emergence, mortality, plant biomass, or seed production in this research indicates that these cultural management practices had a minor effect on Russian thistle suppression. These findings contrast previous research with different weed species in spring barley [13] or spring wheat [25,27,28]. This research does not support using high crop density or narrow row spacing as a broad prescription for Russian thistle suppression. However, spring barley was more competitive with Russian thistle than spring wheat. Including spring barley over spring wheat in a winter wheat–fallow rotation in the PNW is recommended to suppress Russian thistle.

## Figures and Tables

**Table 1:** Effect of year, site, crop, and their interactions on Russian thistle emergence, mortality, seed production, plant biomass, and crop yield (mean ± standard deviation). All means were compared with Tukey’s method.

Factor	Emergence (plants m^−2^)	Mortality (%)	Seed Production (seeds plant^−1^)	Plant Biomass (g)	Crop Yield (kg ha^−1^)
Year	*	ns	ns	ns	***
2018	37.8 (±23.9) a	49.2 (±27.9)	789 (±528)	17.7 (±11.2)	2997 (±1957) b
2019	15.0 (±9.3) b	35.2 (±26.8)	838 (±911)	22.8 (±21.0)	3505 (±1220) a
Site	***	ns	**	ns	***
Pendleton	38.2 (±23.0) a	38.7 (±26.4)	715 (±536) b	13.9 (±7.9)	3811 (±1348) a
Moro	15.1 (±10.6) b	46.1 (±29.6)	910 (±897) a	26.6 (±21.0)	2664 (±1738) b
Crop	*	*	ns	*	ns
SB	24.4 (±20.0) b	47.7 (±30.4) a	604 (±467)	14.4 (±10.2) b	3789 (±1825)
SW	29.4 (±22.5) a	37.0 (±24.7) b	1024 (±897)	26.0 (±20.4) a	2706 (±1228)
Year × Site	***	*	***	*	***
Pendleton_18	55.5 (±20.4) a	51.2 (± 24.9) a	1054 (±540) a	17.1 (±8.3) b	4776 (±1095) a
Pendleton_19	21.4 (±7.7) b	26.2 (±21.7) b	376 (±234) b	10.6 (±6.3) b	2846 (±758) b
Moro_18	20.7 (±10.4) b	47.2 (±30.9) a	516 (±355) b	18.3 (±13.6) b	1218 (±230) c
Moro_19	9.1 (±6.2) c	44.9 (±28.6) a	1331 (±1097) a	34.9 (±24.5) a	4207 (±1235) a
Year × Crop	ns	*	***	**	**
SB_18	34.0 (±23.2)	59.2 (±26.7) a	698 (±528) cb	14.1 (±9.2) b	3493 (±2216) b
SB_19	14.8 (±9.3)	36.1 (±29.8) b	511 (±382) c	14.7 (±11.2) b	4086 (±1296) a
SW_18	41.7 (±24.3)	39.1 (±25.8) b	872 (±522) b	21.2 (±12.0) b	2501 (±1538) c
SW_19	16.2 (±9.5)	34.9 (±23.6) b	1243 (±1161) a	30.8 (±26.1) a	2911 (±752) c
Year × Site × Crop	ns	ns	**	*	***
Pendleton_18_SB	50.2 (±21.0)	64.7 (±20.8)	993 (±544) b	17.7 (±8.4) bc	5611 (±734) a
Pendleton_18_SW	59.8 (±20.3)	37.6 (±21.4)	1114 (±549) b	16.5 (±7.8) bc	3941 (±671) c
Pendleton_19_SB	21.6 (±6.7)	26.7 (±24.1)	345 (±208) c	6.9 (±6.4) c	3308 (±709) c
Pendleton_19_SW	21.2 (±8.9)	25.7 (±19.6)	407 (±261) c	14.4 (±6.2) bc	2384 (±482) d
Moro_18_SB	17.7 (±10.6)	53.7 (±31.1)	402 (±309) c	10.6 (±10.0) bc	1375 (±203) e
Moro_18_SW	23.6 (±10.6)	40.7 (±30.2)	629 (±370) bc	26.0 (±15.1) b	1060 (±124) e
Moro_19_SB	8.0 (±5.8)	45.6 (±32.6)	677 (±447) bc	22.6 (±12.6) bc	4864 (±1294) b
Moro_19_SW	10.5 (±6.6)	44.1 (±24.5)	2078 (±1152) a	46.8 (±25.8) a	3439 (±577) c

Significance indicated by ns *p* > 0.05, * *p* ≤ 0.05, ** *p* ≤ 0.01, and *** *p* ≤ 0.001 for main effects and letters a, b, c, d, and e (α = 0.05) for means separation. SB: spring barley; SW: spring wheat.

**Table 2:** Effect of row spacing (Row) and crop seeding rate (Density) on Russian thistle emergence, mortality, seed production, plant biomass, and crop yield (mean ±standard deviation) in two locations (Pendleton and Moro) over two years (2018 and 2019) with spring barley (SB) and spring wheat (SW). All means were compared with Tukey’s method.

Factor	Emergence (plants m^−2^)	Mortality (%)	Seed Production (seeds plant^−1^)	Plant Biomass (g)	Crop Yield (kg ha^−1^)	Factor	Emergence (plants m^−2^)	Mortality (%)	Seed Production (seeds plant^−1^)	Plant Biomass (g)	Crop Yield (kg ha^−1^)
	Pendleton 2018		SB						SW		
Density						Density					
Low	27.6 (±8.4)	67.0 (±22.9)	1130 (±615)	16.5 (±8.4)	5298 (±919) *	Low	29.8 (±9.2)	34.5 (±23.2)	1240 (±563)	21.4 (±8.7)	3615 (±826) *
High	22.7 (±12.3)	62.3 (±19.8)	856 (±460)	13.8 (±8.8)	5923 (±294) *	High	30.0 (±11.7)	40.7 (±20.6)	988 (±539)	16.6 (±6.6)	4265 (±202) *
Row						Row					
Narrow	19.7 (±7.3)	68.0 (±25.2)	1153 (±361)	17.0 (±7.5)	5652 (±434)	Narrow	26.3 (±8.3)	43.8 (±17.5)	1186 (±582)	20.1 (±9.4)	4165 (±333)
Wide	30.5 (±10.7)	61.4 (±16.3)	834 (±667)	13.3 (±9.5)	5569 (±981)	Wide	33.5 (±11.1)	31.5 (±24.3)	1043 (±542)	17.9 (±6.4)	3715 (±858)
	Moro 2018		SB						SW		
Density						Density					
Low	8.2 (±4.6)	38.9 (±29.3)	479 (±380)	15.8 (±12.5)	1367 (±137)	Low	9.5 (±4.3)	22.0 (±28.5) **	770 (±346)	30.6 (±13.8)	1074 (±156)
High	9.5 (±6.2)	68.5 (±26.9)	326 (±215)	10.5 (±6.5)	1382 (±262)	High	14.2 (±5.4)	59.3 (±18.9) **	488 (±358)	16.3 (±13.3)	1046 (±90)
Row						Row					
Narrow	10.8 (±6.4)	59.9 (±27.7)	384 (±270)	13.3 (±9.7)	1277 (±213) *	Narrow	10.9 (±5.5)	45.2 (±33.9)	591 (±308)	23.7 (±17.6)	1115 (±134)
Wide	6.9 (±3.2)	47.5 (±35.1)	421 (±361)	12.9 (±11.0)	1472 (±144) *	Wide	12.8 (±5.3)	36.2 (±27.6)	668 (±442)	23.3 (±13.2)	1005 (±89)
	Pendleton 2019		SB						SW		
Density						Density					
Low	24.9 (±9.6) *	33.5 (±21.7)	363 (±288)	11.4 (±7.9)	2941 (±316) *	Low	24.0 (±9.6)	31.5 (±21.7)	474 (±288)	13.9 (±7.0)	2487 (±316)
High	18.4 (±7.6) *	19.8 (±16.7)	328 (±228)	7.4 (±3.8)	3674 (±610) *	High	18.5 (±7.6)	19.8 (±16.7)	340 (±228)	9.9 (±4.9)	2281 (±610)
Row						Row					
Narrow	22.9 (±7.8)	22.6 (±22.5)	310 (±229)	8.5 (±6.2)	3485 (±769)	Narrow	26.6 (±9.0) *	22.2 (±19.6)	295 (±150)	9.1 (±3.7)	2417 (±485)
Wide	20.4 (±5.5)	30.8 (±26.5)	380 (±193)	10.3 (±6.7)	3129 (±643)	Wide	15.8 (±4.6) *	29.1 (±20.3)	519 (±306)	14.7 (±7.2)	2350 (±508)
	Moro 2019		SB						SW		
Density						Density					
Low	8.6 (±6.2)	44.2 (±34.4)	739 (±373)	20.5 (±9.4)	5373 (±804) *	Low	11.7 (±6.7)	44.2 (±31.7)	1894 (±1226)	49.9 (±32.7)	3339 (±726) **
High	7.4 (±5.7)	47.0 (±33.0)	614 (±528)	19.6 (±15.8)	4355 (±1533) *	High	9.3 (±6.9)	43.9 (±17.3)	2263 (±1136)	49.1 (±19.2)	3574 (±403) **
Row						Row					
Narrow	6.2 (±5.3)	34.9 (±33.8)	643 (±551)	18.0 (±13.9)	4590 (±1690)	Narrow	5.4 (±4.3) *	32.3 (±27.4)	1858 (±1238)	42.4 (±17.2)	3138 (±651)
Wide	9.8 (±6.1)	56.2 (±29.6)	711 (±347)	22.1 (±11.6)	5137 (±749)	Wide	15.6 (±3.7) *	55.8 (±15.1)	2298 (±1108)	56.6 (±32.0)	3775 (±248)

Significance indicated by *p* ≤ 0.1, * *p* ≤ 0.05, and ** *p* ≤ 0.01.

**Table 3:** Comparison of crop yield with and without Russian thistle pressure for each location and crop in 2019.

Site	Crop	*p*-Value
Pendleton	Spring wheat	0.374
	Spring barley	0.820
Moro	Spring wheat	0.700
	Spring barley	0.219

## Data Availability

The data presented in this study are openly available at the ScholarArchive@OSU (https://ir.library.oregonstate.edu/concern/datasets/r781wp412).
